# Orexin Neurons Contribute to Central Modulation of Respiratory Drive by Progestins on *ex vivo* Newborn Rodent Preparations

**DOI:** 10.3389/fphys.2019.01200

**Published:** 2019-09-27

**Authors:** Camille Loiseau, Alexis Casciato, Besma Barka, Florence Cayetanot, Laurence Bodineau

**Affiliations:** Institut National de la Santé et de la Recherche Médicale, UMR_S1158 Neurophysiologie Respiratoire Expérimentale et Clinique, Sorbonne Université, Paris, France

**Keywords:** CO_2_/H^+^ chemosensitivity, congenital central hypoventilation syndrome, etonogestrel, *ex vivo* central nervous system preparation, orexin, progestin

## Abstract

Dysfunction of central respiratory CO_2_/H^+^ chemosensitivity is a pivotal factor that elicits deep hypoventilation in patients suffering from central hypoventilation syndromes. No pharmacological treatment is currently available. The progestin desogestrel has been suggested to allow recovery of respiratory response to CO_2_/H^+^ in patients suffering from central hypoventilation, but except the fact that supramedullary regions may be involved, mechanisms are still unknown. Here, we tested in neonates whether orexin systems contribute to desogestrel’s central effects on respiratory function. Using isolated *ex vivo* central nervous system preparations from newborn rats, we show orexin and almorexant, an antagonist of orexin receptors, supressed strengthening of the increase in respiratory frequency induced by prolonged metabolic acidosis under exposure to etonogestrel, the active metabolite of desogestrel. In parallel, almorexant suppressed the increase and enhanced increase in *c-fos* expression in respiratory-related brainstem structures induced by etonogestrel. These results suggest orexin signalisation is a key component of acidosis reinforcement of respiratory drive by etonogestrel in neonates. Although stage of development used is different as that for progestin clinical observations, presents results provide clues about conditions under which desogestrel or etonogestrel may enhance ventilation in patients suffering from central hypoventilation syndromes.

## Introduction

Elaboration of a central respiratory drive (CRD) able to match both metabolic demand for O_2_ and clearance of CO_2_ relies on central and peripheral structures that generate, integrate, encode, and convey relevant information from the entire body (Feldman et al., 2003). Central respiratory chemosensitivity refers to ability of cellular and molecular sensors to detect CO_2_/H^+^ variations within brain and initiate appropriate adjustments in ventilation (Guyenet and Bayliss, 2015). The *paired-like homeobox 2b* (*phox2b)*-expressing neurons of retrotrapezoid nucleus (RTN) are considered as an important site of CO_2_/H^+^ chemosensitivity, especially during the neonatal period (Mulkey et al., 2004; Dubreuil et al., 2008). Additionally, there is a general agreement that other neuronal structures, distributed along brainstem and diencephalon contribute to the respiratory response to CO_2_/H^+^ challenges (Biancardi et al., 2008; Lopes et al., 2012; Song et al., 2012).

Central hypoventilation syndromes (CHS) are neurorespiratory diseases that result from dysfunction of sensory receptors, respiratory rhythm generators, or other central respiratory structures (Cielo and Marcus, 2014). Among these diseases, congenital central hypoventilation syndrome (CCHS) is the best documented. CCHS is a life-threatening sleep-related hypoventilation syndrome associated with an absent or blunted respiratory response to CO_2_/H^+^ (Weese-Mayer et al., 2010). The *PHOX2B* gene is the disease-defining gene for CCHS (Amiel et al., 2003). Loss of respiratory response to CO_2_/H^+^ is caused, at least partially, by dysfunction of CO_2_/H^+^ chemosensitive PHOX2B-positive neurons of the RTN (Dubreuil et al., 2008; Amiel et al., 2009). No pharmacological treatment for CCHS is available, but a serendipitous finding revealed two adult women with CCHS recovered CO_2_/H^+^ chemosensitivity concomitant with oral consumption for contraceptive purpose of desogestrel, a potent synthetic progestin belonging to gonane family (Schindler et al., 2003; Straus et al., 2010). Progesterone and synthetic progestins exert a stimulatory effect on respiratory adaptation to gas challenges and baseline respiratory drive in both humans and animal models (Bayliss et al., 1987, 1990; Slatkovska et al., 2006; Joubert et al., 2016). It has thus been suggested desogestrel may have been involved in recovery of CO_2_/H^+^ chemosensitivity in CCHS. However, this recovery may not be systematic, as suggested by the non-improvement of respiratory response to CO_2_/H^+^ observed in a CCHS patient deliberately given desogestrel (Li D.C. et al., 2013). This discrepancy may rely on idiosyncrasy such as integrity or functioning of one or more central structures. This possibility is supported by the fact that some CCHS patients present alterations in certain central structures that have been suggested in connection with hypoxic episodes (Harper et al., 2014). This context highlights the pressing necessity to elucidate complex mechanisms involved in this gonane progestin effect.

We hypothesized desogestrel, or rather its biologically active metabolite 3-ketodesogestrel (etonogestrel, ETO) (Verhoeven et al., 1998), is able to induce the recovery of CO_2_/H^+^ chemosensitivity in some CCHS patients by activating, or enhancing activation, of CO_2_/H^+^ sensitive central structures still functional in CCHS patients. Elucidating a physiological basis that underlies recovery of ETO-induced CO_2_/H^+^ chemosensitivity is a crucial prerequisite to further evaluate conditions under which it could constitute a therapeutic track for the treatment of CCHS and even CHS in general. In a first exploratory study, we demonstrated acute exposure to ETO potentiates an increase in respiratory frequency (f_R_) induced by metabolic acidosis through a mechanism involving supramedullary encephalic regions but involved cell population(s) which remain unknown (Loiseau et al., 2014).

The present study was designed to decipher central mechanisms implicated in strengthening of respiratory response to metabolic acidosis induced by ETO. First, we demonstrate the diencephalon is essential to enhancement of respiratory response to prolonged metabolic acidosis by the progestin in *ex vivo* central nervous system (CNS) preparations from newborn rats. Second, pattern of *c-fos* expression, an effective marker of neuronal activation, revealed strengthening of respiratory responses to prolonged metabolic acidosis by ETO is associated with activation or enhanced activation of brainstem respiratory structures. Finally, we demonstrate diencephalic orexin neurons constitute a key neuronal population in the effect of ETO, because blocking orexin signaling resulted in loss of both strengthening of respiratory response to prolonged metabolic acidosis and activation or enhanced activation of brainstem respiratory structures, except medullary raphe nuclei.

## Materials and Methods

Experiments were performed on both male and female newborn Sprague-Dawley rats (0–3 days old, 8.1 ± 0.06 g (mean ± standard error of the mean), Janvier Labs; Le Genest Saint Isle, France) in accordance with Directive 2010/63/EU of the European Parliament and of the Council of 22 September, 2010 and French law (2013/118). All protocols were approved by Charles Darwin Ethics Committee for Animal Experimentation (Ce5/2011/05; APAFIS#2210-2015100812195835v2).

### Drugs

The following drugs were used: dimethyl sulfoxide (DMSO, Sigma-Aldrich, Saint-Quentin Fallavier, France), ETO (3-ketodesogestrel, Sigma-Aldrich, Saint-Quentin Fallavier, France) prepared in DMSO, orexin A (O6012, Sigma-Aldrich, Saint-Quentin-Fallavier, France) and almorexant (ACT-078573, (2R) - 2 - {(1S) - 6,7 – dimethoxy - 1 - [2 - (4 - trifluoromethylphenyl) - ethyl] - 3,4 – dihydro - 1Hisoquinolin-2-yl} - *N*-methyl – 2 - phenyl-acetamide), both prepared in saline.

### *Ex vivo* Preparations of Isolated Central Nervous System

Medullary-spinal cord (MS, *n* = 35), ponto-medullary-spinal cord (PMS, *n* = 30), brainstem-spinal cord (BS, *n* = 29), and diencephalon-brainstem-spinal cord (DBS, *n* = 257) *ex vivo* preparations were isolated under deep cold anesthesia by immersion in ice water (Danneman and Mandrell, 1997), as previously described (Suzue, 1984; Okada et al., 1998; Loiseau et al., 2014). In all cases, a caudal section was made between the seventh and eighth cervical spinal nerve roots. Among the various types of preparations, the level of the rostral section differed: at the level of the anterior inferior cerebellar arteries just caudal to the VIII cranial nerve exit points for MS preparations, rostral to the fifth cranial nerves at the level of the superior cerebellar arteries and caudal edge of the inferior colliculi for PMS preparations, at the level of the intersection between the posterior cerebral and posterior communicating arteries for BS preparations, and at the level of the rostral extremity of the optic chiasm for DBS preparations.

Preparations were superfused with aCSF (in mM: 129 NaCl, 3.35 KCl, 1.15 MgCl_2_, 0.58 NaH_2_PO_4_, 30 D-glucose, 1.26 CaCl_2_, and NaHCO_3_ at various concentrations depending on the experimental condition (Murakoshi et al., 1985)) maintained at 26 ± 1°C, saturated with O_2_, and adjusted to the appropriate pH by bubbling with 95% O_2_ and 5% CO_2_. As molecular detectors sensing H^+^ and CO_2_ changes are described as sensitive to an increased concentration of H^+^, we performed pH variation of aCSF to mimic physiological consequences of an increase in CO_2_ (Gestreau et al., 2010; Song et al., 2012; Guyenet and Bayliss, 2015; Kumar et al., 2015). It should be noted, however, that future experiments carried out under conditions of acidosis induced by a rise in CO_2_ could reveal additional mechanisms to those observed in the study. Normal pH-aCSF (pH 7.4) and metabolic acidosis-aCSF (pH 7.23) differed in terms of NaHCO_3_ concentration (21 and 15 mM, respectively) (Suzue, 1984; Murakoshi et al., 1985; Gestreau et al., 2010; Loiseau et al., 2014). Electrical activity of a fourth cervical ventral nerve root (C4) was recorded using a suction electrode, filtered (300–1000 Hz), amplified (×10000; Differential AC Amplifier Model 1700; A-M systems), integrated (time constant 100 ms), and digitized through a PowerLab 4SP, with a sampling frequency of 2500 Hz, and visualized and analyzed using LabChart 7.2 data acquisition and analysis software (ADInstruments, Castle Hill, Australia). As previously reported, f_R_ was defined as the burst frequency recorded from C4 for 1 min (burst⋅min^–1^) (Suzue, 1984; Loiseau et al., 2014; Joubert et al., 2016). We did not observe any qualitative changes in integrated C4 burst activity, which was regarded as an index of inspiratory activity, in a first set of experiments, consistent with previous observations (Straus et al., 2010; Joubert et al., 2016). We thus focussed our analysis on f_R_.

### Experimental Protocols

After surgery, all preparations were left to stabilize for 30 min in normal pH-aCSF. Baseline values were defined as mean value during the last 10 min of stabilization period. After that, a given preparation was only exposed to a given pharmacological protocol described in the following paragraphs. In agreement with previous reports concerning ETO or other steroids respiratory effect on newborn rodent less than 4 days old, we pooled data obtained from male and female *ex vivo* preparations (Ren and Greer, 2006; Loiseau et al., 2014; Joubert et al., 2016).

#### Determination of the Effect of Etonogestrel on Respiratory Response to Prolonged Metabolic Acidosis of *ex vivo* Preparations

After stabilization, preparations were superfused for 15 min with normal pH-aCSF, without drugs (MS preparation, *n* = 10; PMS preparations, *n* = 10; BS preparations, *n* = 10 and DBS preparations, *n* = 9) or supplemented with either DMSO (at 0.01%, MS preparation, *n* = 16; PMS preparations, *n* = 10; BS preparations, *n* = 9 and DBS preparations, *n* = 13) or ETO in 0.01% DMSO at 5⋅10^–2^ μM (DBS preparation, *n* = 10), 5⋅10^–1^ μM (MS preparation, *n* = 9; PMS preparations, *n* = 10; BS preparations, *n* = 10 and DBS preparations, *n* = 13), 1 μM (DBS preparations, *n* = 9) or 2 μM (DBS preparations, *n* = 12). Mean f_R_ value obtained during the last 5 min of this period was called pre-metabolic acidosis value. Then, preparations were superfused with metabolic acidosis-aCSF without drugs or supplemented with either DMSO or ETO for 30 min. Calculated mean f_R_ during metabolic acidosis exposure was called metabolic acidosis value and was expressed relative to pre-metabolic acidosis value.

As a control, preparations were superfused, after stabilization, with either DMSO (0.01%, *n* = 12) or 5⋅10^–1^ μM ETO (*n* = 24) in normal pH condition for 30 min.

#### Determination of the Potential Involvement of Orexin Systems in the Effect of Etonogestrel on Respiratory Response to Prolonged Metabolic Acidosis

##### Determination of concentrations of orexin and almorexant, an OX1R and OX2R antagonist

The effect of orexin on f_R_ at various concentrations on DBS preparations was analyzed to determine both orexin concentration for observing an effect on the central respiratory drive in these *ex vivo* preparations as previously reported on more reduced *ex vivo* preparations (Sugita et al., 2014; Umezawa et al., 2015) and concentration of antagonist (almorexant) that is necessary and sufficient to suppress the effect of orexin. Based on previous studies, orexin A was applied at 5⋅10^–4^ μM (*n* = 6), 10^–4^ μM (*n* = 6), 10^–3^ μM (*n* = 10), 10^–2^ μM (*n* = 11) and 10^–1^ μM (*n* = 5) (Sugita et al., 2014; Umezawa et al., 2015). f_R_ was measured during 1 min after 15 min of exposure and expressed relative to baseline values (Sugita et al., 2014; Umezawa et al., 2015).

We examined the effect of 5⋅10^–2^ μM (*n* = 8), 5⋅10^–1^ μM (*n* = 7), and 5 μM (*n* = 9) almorexant on f_R_ of DBS preparations at normal pH, based on the literature (Brisbare-Roch et al., 2007). The effect on f_R_ was assessed by comparing f_R_ during 1 min after 15 min of exposure to baseline values.

On the basis of the results obtained in the two series which have just been described, we applied almorexant and orexin A (10^–2^ μM, *n* = 8) together to DBS preparations to determine whether 5⋅10^–2^ μM almorexant completely blocked orexin signaling. After stabilization, preparations were preincubated with normal pH-aCSF containing almorexant for 15 min before being exposed to normal pH-aCSF containing both almorexant and orexin A for 15 min. The effect of orexin A exposure in the presence of almorexant was assessed by comparing f_R_ calculated over the last min of period of co-application to both baseline values and values obtained under orexin A alone.

##### Analysis of the effect of etonogestrel on the increase in f_R_ induced by prolonged metabolic acidosis in the presence of orexin or almorexant

We evaluated the effect of ETO on respiratory response to prolonged metabolic acidosis under antagonization of orexin signaling by co-applying 5⋅10^–2^ μM almorexant and DMSO (*n* = 13) and 5⋅10^–2^ μM almorexant and 5⋅10^–1^ μM ETO (*n* = 10). After stabilization, DBS preparations were exposed for 15 min to almorexant in normal pH-aCSF. Preparations were then superfused for 15 min with normal pH-aCSF containing almorexant along with either DMSO or ETO. This period was followed by 30 min of superfusion with metabolic acidosis-aCSF containing the same pharmacological agents i.e., almorexant and DMSO or almorexant and ETO. The effect on f_R_ of co-application almorexant/DMSO or almorexant/ETO in normal pH condition was assessed by comparing f_R_ calculated during the last 5 min of these exposures, i.e., pre-metabolic acidosis values, to baseline values. The effect of ETO on prolonged metabolic acidosis response under almorexant was assessed by comparing f_R_ calculated during metabolic acidosis period to pre-metabolic acidosis value.

The effect of orexin alone on the increase in f_R_ induced by prolonged metabolic acidosis was appreciated by applying 10^–2^ μM orexin (*n* = 6). After stabilization, DBS preparations were maintained during 30 min under normal pH-aCSF containing 0.01% DMSO and then exposed during 30 min to metabolic acidosis-aCSF containing 10^–2^ μM orexin with 0.01% DMSO. Prolonged metabolic acidosis response under orexin was assessed by comparing f_R_ calculated during metabolic acidosis period to pre-metabolic acidosis value.

Finally, we evaluated the effect of ETO on respiratory response to prolonged metabolic acidosis under orexin signaling by two experimental designs. First, after stabilization, DBS preparations were maintained during 30 min under normal pH-aCSF containing 0.01% DMSO and then exposed during 30 min to metabolic acidosis-aCSF containing 10^–2^ μM orexin and 5⋅10^–1^ μM ETO with 0.01% DMSO (*n* = 10). Second, after stabilization, DBS preparations were exposed for 15 min to orexin in normal pH-aCSF containing 0.01% DMSO (*n* = 10). Preparations were then superfused for 15 min with normal pH-aCSF containing orexin and ETO. This period was followed by 30 min of superfusion with metabolic acidosis-aCSF containing orexin and ETO.

### Immunohistochemistry

#### Immunohistochemical Procedures

Immunohistochemical detection of c-FOS was performed on DBS preparations exposed to normal pH or prolonged metabolic acidosis conditions with DMSO alone (*n* = 4 and *n* = 4, respectively) or 5⋅10^–1^ μM ETO (*n* = 12 and *n* = 12, respectively) after having undergone the same protocols as those previously described in the relevant paragraphs. Preparations were fixed by immersion in 4% paraformaldehyde in 0.1 M phosphate buffer (pH 7.4) for 48 h at 4°C after exposure to DMSO or 5⋅10^–1^ μM ETO with or without almorexant. All preparations were left to stabilize for 30 min in normal pH-aCSF after surgery. For preparations not exposed to almorexant, either they were maintained under normal pH conditions with DMSO alone or 5⋅10^–1^ μM ETO during 30 min or they were superfused with aCSF containing DMSO alone or 5⋅10^–1^ μM ETO during 15 min under normal pH following by 30 min under metabolic acidosis conditions. For preparations exposed to almorexant, they were exposed for 15 min to almorexant in normal pH-aCSF, then for 15 min with normal pH-aCSF containing almorexant along with either DMSO or 5⋅10^–1^ μM ETO, and finally, for 30 min with metabolic acidosis-aCSF containing the same pharmacological agents i.e., almorexant and DMSO or almorexant and 5⋅10^–1^ μM ETO. After immersion in paraformaldehyde solution, preparations were cryoprotected for 72 h in 0.1 M phosphate buffer containing 30% sucrose. Immunohistochemical detection of c-FOS was processed on free-floating coronal sections (40 μm) by incubation with first, a rabbit polyclonal antibody against c-FOS (sc-253; Santa Cruz Biotechnology Inc., CA, United States, 1:10000, in 1% bovine serum albumin, BSA; 48 h, 4°C), second a biotinylated goat anti-rabbit IgG antibody (Vector Laboratories, Burlington, Canada, 1:500; 2 h), and finally an avidin-biotin-peroxidase complex (ABC; Novostain Super ABC kit, Novocastra Laboratories, Newcastle, United Kingdom, 1:250; 1 h). Peroxidase activity was detected using a solution containing 0.02% 3,3′- diaminobenzidine tetrahydrochloride, 0.04% nickel ammonium sulfate and 0.01% H_2_O_2_ in 0.05 M Tris–HCl buffer (pH 7.6), which results in a blue/gray chromogen.

Dual detection of c-FOS and orexin A was performed on DBS preparations exposed to prolonged metabolic acidosis conditions under DMSO alone, 5⋅10^–1^ μM ETO alone, almorexant/DMSO, or almorexant/5⋅10^–1^ μM ETO to specifically analyse changes in cell activity in orexin neurons. After c-FOS detection, sections were incubated with a goat polyclonal antibody against orexin A (sc-8070, Santa Cruz Biotechnology Inc., CA, United States, 1:2000, in 1% BSA; 48 h, 4°C), with a biotinylated rabbit anti-goat IgG antibody (Vector Laboratories, Burlington, Canada, 1:500; 2 h) and then with ABC (1:250; 1 h). Peroxidase activity was detected with 0.02% 3,3′-diaminobenzidine tetrahydrochloride and 0.01% hydrogen peroxide in 0.05 M Tris–HCl buffer (pH 7.6) resulting in a brown chromogen.

For single or dual detections, control sections were processed in parallel, but with the omission of primary or secondary antibodies. No labeling was observed on control sections.

All sections were mounted in sequential caudo-rostral order on silanized slides, air-dried, and coverslipped with Entellan (VWR, International S.A.S).

#### Cell Counting

c-FOS and c-FOS/orexin immunolabeled cells were visually counted, by an investigator blinded to the samples under a light microscope (Leica DM 2000; Leica Microsystems, Heidelberg, Germany) at high magnification (×200 or ×400 depending on the immunolabelled density of cells) by varying micrometer of the microscope which was essential for tissue sections of 40 micrometers. Analyzed structures involved in CRD elaboration or adaptation, were localized using standard landmarks (Paxinos, 1998). Microphotographs were captured with a digital camera (Leica DFC450C, Leica Microsystems, Heidelberg, Germany). We counted c-FOS positive cells along *medulla oblongata* in commissural, medial and ventrolateral parts of the nucleus of the solitary tract (cNTS, mNTS, and vlNTS), in parapyramidal area (PP), at the lateral edge of pyramidal tract, extending from pyramidal decussation to caudal edge of the facial nucleus (Voituron et al., 2006), in medullary raphe nuclei i.e., raphe pallidus, obscurus and magnus nuclei (RPa, ROb, and RMg), in RTN (in the ventral medullary surface in ventromedial position under the facial nucleus) (Dubreuil et al., 2009), in pFRG (in the ventral medullary surface in ventrolateral position under the facial nucleus) (Onimaru and Homma, 2003), and in ventrolateral reticular nucleus of the medulla (VLM), a neuronal column ventral to nucleus ambiguus, extending from pyramidal decussation to caudal edge of facial nucleus that encompass the preBotC and the A1/C1 group of neurons. In pons, c-FOS positive cells were counted in locus coeruleus (LC), A5 region (A5), lateral subnucleus of parabrachial nucleus (lPB), and medial subnucleus of the parabrachial nucleus/kölliker-fuse nucleus (mPB/KF). In mesencephalon, c-FOS positive cells were counted in dorsal, dorsolateral, lateral and ventrolateral parts of the periaqueductal gray (dPAG, dlPAG, lPAG, and vlPAG). In diencephalon, single-labeled (c-FOS) or double-labeled (c-FOS/orexin) cells were counted in lateral hypothalamic area (LH), posterior hypothalamic area (PH), dorsomedial hypothalamic nucleus (DMH), and perifornical region (PeF). The mean number of c-FOS and c-FOS/orexin positive cells per section were quantified for each structure.

### Statistics

Data were analyzed with GraphPad (GraphPad Prism8, San Diego, CA, United States). Normality of data distribution for f_R_, c-FOS and c-FOS/orexin values was assessed using d’Agostino and Pearson omnibus normality test. Baseline f_R_ are expressed as mean ± standard error of the mean as data distribution is normal. As electrophysiological and histological series include group(s) with a non-normal distribution, all results are expressed as a median and interquartile range [Q1; Q3]. Parametric or non-parametric tests were performed, depending on normality. Within each group, the effect of an experimental condition on f_R_ was assessed using a one-sample *t* test or a Wilcoxon signed rank test. Comparisons of the effect on f_R_ between two groups were performed using two-tailed unpaired *t* tests or Mann Whitney tests. To highlight differences induced by experimental conditions between more than two groups, intergroup comparisons of f_R_ or percentage of change of f_R_ were performed using a one-way ANOVA, followed by Bonferroni’s multiple comparison test, or Kruskal-Wallis test followed by Dunn’s or Benjamini, Krieger and Yekutieli’s multiple comparison test. Number of c-FOS positive cells and c-FOS/orexin cells per section were compared between two conditions using one-tailed or two-tailed Mann Whitney tests, depending on stated hypothesis. Differences were considered to be significant if *p* < 0.05.

## Results

### Baseline Respiratory Frequency of *ex vivo* Preparations Used for All Respiratory Drive Analyses

Baseline f_R_ was 7.19 ± 0.21 bursts⋅min^–1^ for DBS (*n* = 221), 5.47 ± 0.59 bursts⋅min^–1^ for BS (*n* = 29), 4.51 ± 0.50 bursts⋅min^–1^ for PMS (*n* = 30), and 8.87 ± 0.30 bursts⋅min^–1^ for MS (*n* = 35) preparations. f_R_ of all the 221 DBS preparations, as well as f_R_ of 4 different random selections of DBS preparations among the 221 (7.16 ± 0.60; 7.29 ± 0.62; 7.17 ± 0.55; 7.31 ± 0.67 bursts⋅min^–1^) was significantly higher than f_R_ of BS (*p* < 0.04) and PMS (*p* < 0.001) preparations but significantly lower than that of MS preparations (*p* < 0.05).

### Etonogestrel Strengthens Respiratory Response to Prolonged Metabolic Acidosis in a Small Concentration Range

**FIGURE 1:**
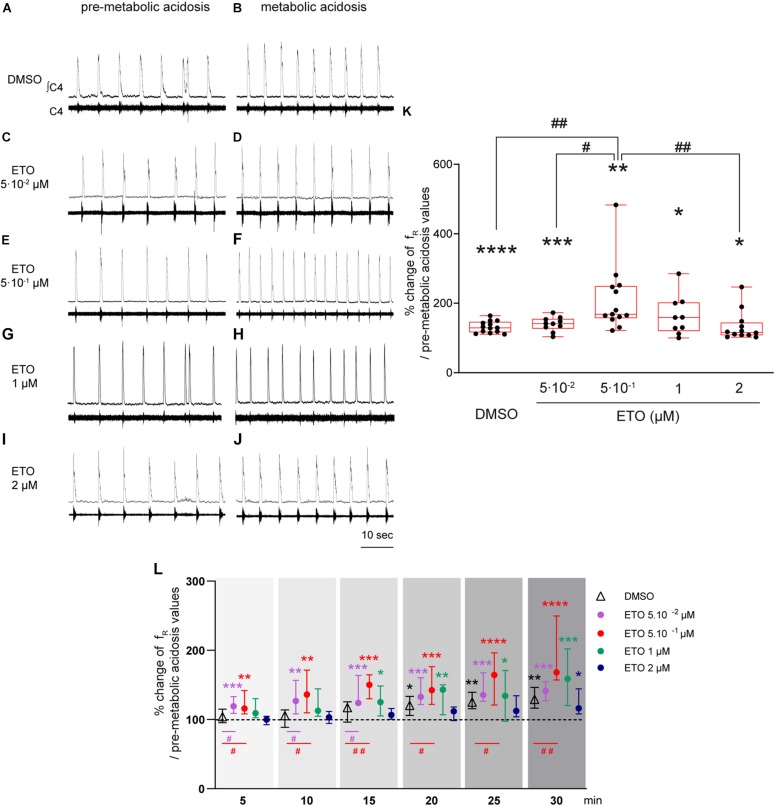
Etonogestrel strengthens respiratory response to prolonged metabolic acidosis at a specific concentration. **(A–J)** Representative traces illustrating the respiratory C4 activity recorded in DBS preparations during condition of pre-metabolic acidosis under DMSO **(A)** or ETO **(C,E,G,I)** exposure and during condition of prolonged metabolic acidosis under DMSO **(B)** or ETO **(D,F,H,J)** exposure. Note during pre-metabolic condition, baseline f_R_ of all preparations were similar suggesting ETO did not change baseline f_R_ on these DSB preparations as we previously reported Loiseau et al. (2014). **(K)** Column scatter graph with a superimposed box and whisker plot (median [Q1;Q3]) illustrating percentage of change in f_R_ at 25–30 min of metabolic acidosis under conditions of prolonged metabolic acidosis in the presence of either DMSO (white bar, *n* = 13) or ETO (gray bars) at 5⋅10^– 2^ (*n* = 10), 5⋅10^– 1^ (*n* = 13), 1 (*n* = 9), or 2 μM (*n* = 12). ^∗^ Indicates a significant intragroup change (one-sample *t* test) in f_R_ relative to values obtained just prior to placing under conditions of metabolic acidosis. ^#^ Indicates a significant difference (one-way ANOVA followed by Bonferroni’s multiple comparison test) between groups. ^∗^*p* < 0.05, ^∗∗^*p* < 0.01, ^∗∗∗^*p* < 0.001, ^∗∗∗^*p* < 0.0001, ^#^*p* < 0.05, ^##^*p* < 0.01. **(L)** Median with interquartile range of f_R_ in percentage of pre-metabolic acidosis values for DMSO (white triangle), ETO at 5⋅10^– 2^ (purple circle), 5⋅10^– 1^ (red circle), 1 (green circle), and 2 (blue circle) μM by 5 min period throughout metabolic acidosis exposure. ^∗^Indicates a significant intragroup change (Kruskal-Wallis test followed by Dunn’s multiple comparison test) in f_R_ relative to values obtained just prior to placing under conditions of metabolic acidosis. ^#^Indicates a significant difference (Kruskal-Wallis test followed by two-stage linear step-up procedure of Benjamini, Krieger and Yekutieli multiple comparison test) between groups. ^∗^*p* < 0.05, ^∗∗^*p* < 0.01, ^∗∗∗^*p* < 0.001, ^∗∗∗^*p* < 0.0001, ^#^*p* < 0.05, ^##^*p* < 0.01. ∫C4: integrated activity of the C4 ventral nerve root; C4: electrical activity of the C4 ventral nerve root; ETO: etonogestrel.

In response to prolonged metabolic acidosis, DBS preparations superfused without drugs or exposed to DMSO exhibit a similar significant increase in f_R_ (+25.0% [16.6;40.7], *p* < 0.01 and +29.2% [16.4;46.4], *p* < 0.0001, respectively at 25–30 min of metabolic acidosis). In preparations exposed to 5⋅10^–2^ μM, 1 μM, and 2 μM ETO, increase in f_R_ induced by prolonged metabolic acidosis (+41.3% [27.0;54.8], *p* < 0.001, +58.8% [20.1;102.1.8], *p* < 0.05, and +16.2% [8.2;44.3], *p* < 0.05, respectively at 25–30 min of metabolic acidosis) is not significantly different from that observed in preparations exposed to DMSO (Figure 1). In contrast, increase in f_R_ induced by prolonged metabolic acidosis in the presence of 5⋅10^–1^ μM ETO (+68.2% [57.1;149.3], *p* < 0.01 at 25–30 min of metabolic acidosis) is significantly greater than that observed in preparations exposed to DMSO (*p* < 0.01 at 25–30 min of metabolic acidosis) (Figure 1). This strengthening of increase in f_R_ is also significantly greater than that observed for preparations exposed to 5⋅10^–2^ and 2 μM ETO (*p* < 0.05 and *p* < 0.01, respectively at 25–30 min of metabolic acidosis) (Figure 1). Note ETO at 5⋅10^–1^ μM does not induce a significant increase in f_R_ at normal pH (DMSO: (+6.9% [−26.1;75.9] vs. ETO: +28.7% [15.0;50.0]).

In addition to comparison of last 5 min of metabolic acidosis, analysis of f_R_ modifications observed over 30 min of exposure reveals differences in the time required to observe an increase in f_R_ induced by metabolic acidosis (Figure 1). Under DMSO exposure, f_R_ increases significantly from 15–20 min exposure to metabolic acidosis (+20.3% [5.9;33.5], *p* < 0.05). Under ETO at 5⋅10^–2^ and 5⋅10^–1^ μM, f_R_ increases significantly more precociously than without ETO, from the first 5 min of metabolic acidosis (+19.1% [8.8;33.2], *p* < 0.0005 and +15.8% [8.3;41.9], *p* < 0.003, respectively). For ETO at 0.05 μM, f_R_ is significantly greater than that observed in preparations exposed to DMSO from the first 15 min (*p* < 0.05). For ETO at 0.5 μM, f_R_ is significantly greater than that observed in preparations exposed to DMSO throughout metabolic exposure (*p* < 0.05). Under ETO at 1 μM, f_R_ increases significantly from 10–15 min of metabolic acidosis (+25.0% [5.1;48.4], *p* < 0.03). Under ETO at 2 μM, f_R_ increases significantly from 25–30 min of metabolic acidosis (+16.2% [8.2;44.3], *p* < 0.03). For ETO at 1 and 2 μM, f_R_ is not significantly different from that observed under DMSO exposure throughout metabolic exposure.

In light of these results, subsequent experiments involving ETO were performed in the presence of 5⋅10^–1^ μM, the effective dose of this progestin.

### Etonogestrel-Dependant Strengthening of Respiratory Response to Prolonged Metabolic Acidosis Requires Presence of the Diencephalon

**FIGURE 2:**
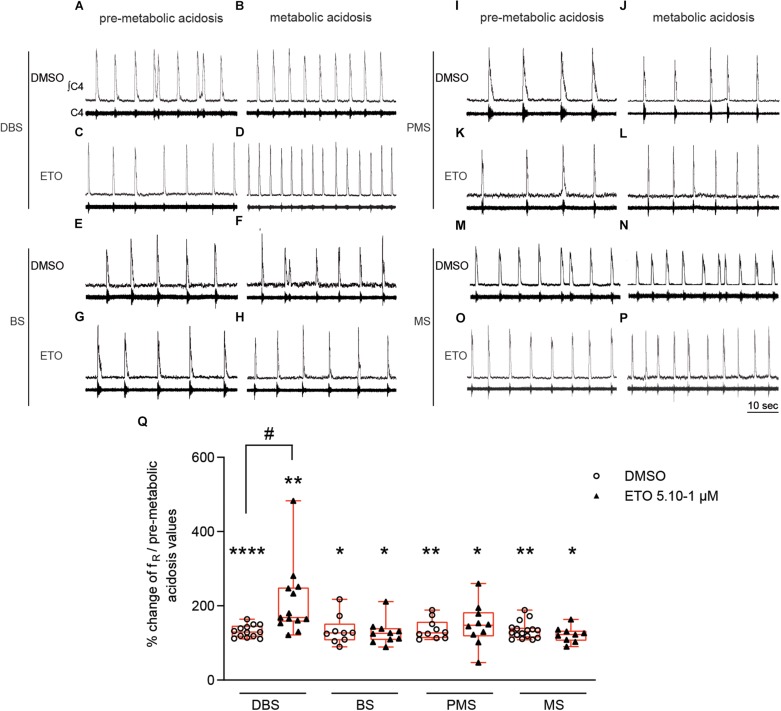
Strengthening of respiratory response to prolonged metabolic acidosis by etonogestrel depends on the presence of diencephalon. **(A–P)** Representative traces illustrating respiratory C4 activity recorded in DBS **(A–D)**, BS **(E–H)**, PMS **(I–L)**, and MS **(M–P)** preparations, during condition of pre-metabolic acidosis under DMSO **(A,E,I,M)** or ETO **(C,G,K,O)** exposure and during condition of prolonged metabolic acidosis under DMSO **(B,F,J,N)** or ETO **(D,H,L,P)** exposure. **(Q)** Column scatter graph with a superimposed box and whisker plot (median [Q1;Q3]) showing percentage of change of f_R_ at 25–30 min of metabolic acidosis under conditions of prolonged metabolic acidosis in the presence of either DMSO or ETO on DBS (*n* = 13 and *n* = 13, respectively), BS (*n* = 9 and *n* = 10, respectively), PMS (*n* = 10 and *n* = 10, respectively), and MS (*n* = 16 and *n* = 9, respectively) preparations. ^∗^Indicates a significant intragroup change (one-sample *t* test) in f_R_ relative to the values obtained just prior placing under conditions of metabolic acidosis. #Indicates a significant difference (one-way ANOVA followed by Bonferroni’s multiple comparison test) between groups. ^∗^*p* < 0.05, ^∗∗^*p* < 0.01, ^****^p < 0.0001, ^#^*p* < 0.05. ∫C4: integrated activity of the C4 ventral nerve root; C4: electrical activity of the C4 ventral nerve root; ETO: etonogestrel, DBS: diencephalon-brainstem-spinal cord preparation, BS: brainstem-spinal cord preparation, PMS: ponto-medullary-spinal cord preparation, MS: medullary-spinal cord preparation.

Prolonged metabolic acidosis, without drug exposure, induces significant increases in f_R_ in BS (+22.5% [0.0;38.1], *p* < 0.01), PMS (+15.5% [0.0;21.3], *p* < 0.05), and MS (+29.2% [1.8;33.3], *p* < 0.05) preparations at 25–30 min of metabolic acidosis, as in DBS preparations. These increases are equivalent to those observed under DMSO (BS: +27.3% [6.8;52.2], *p* < 0.05; PMS: +27.0% [12.9;57.1], *p* < 0.01; MS: +30.9 [14.9;41.0], *p* < 0.01) at 25–30 min of metabolic acidosis (Figure 2). Comparison of increases in f_R_ for all types of preparations (DBS, BS, PMS, and MS) showed respiratory response to prolonged metabolic acidosis in the presence of DMSO is of same magnitude, irrespective of the rostral extension of preparations (Figure 2). In addition, ETO does not strengthen respiratory response to prolonged metabolic acidosis in preparations lacking diencephalic structures at concentration at which it produces such an effect in DBS preparations (5⋅10^–1^ μM). Indeed, BS, PMS, and MS preparations displayed significant increases in f_R_ in response to prolonged metabolic acidosis in the presence of ETO (BS: +25.9% [8.1;39.8], *p* < 0.05; PMS: +47.5% [17.6;83.1], *p* < 0.05; MS +22.5% [5.9;33.5], *p* < 0.05) at 25–30 min of metabolic acidosis, which are similar to those observed in their respective control groups exposed to DMSO (Figure 2).

### Etonogestrel Modifies *c-fos* Expression Induced by Prolonged Metabolic Acidosis in Central Structures Involved in Elaboration of the Central Respiratory Drive and/or Its Regulation

#### Etonogestrel Does Not Induce Changes in *c-fos* Expression in Normal pH Condition

We first quantified c-FOS positive cells in DBS preparations superfused with either DMSO or ETO (5⋅10^–1^ μM) at normal pH to determine potential ETO-induced changes of cell activity that do not depend on stimulation of metabolic acidosis. There is no significant difference in number of c-FOS-positive cells between DMSO or ETO exposed preparations (Table 1), in accordance with the fact that f_R_ is not significantly modified by ETO at normal pH.

**TABLE 1:** *c-fos* expression in brainstem and diencephalic structures of DBS *ex vivo* preparations under normal pH and prolonged metabolic acidosis conditions in the presence of either DMSO or etonogestrel.

		**Normal pH**		**Prolonged metabolic acidosis**	
		
		**DMSO**	**ETO**	**DMSO**	**ETO**
		**(*n* = 4)**	**(*n* = 4)**	**(*n* = 8)**	**(*n* = 8)**
Medulla oblongata	cNTS	28.2 [21.4;42.4]	39.0 [18.2;51.0]	16.7 [12.6;24.0]	73.3 [55.2;88.3]^###,§^
	vlNTS	1.5 [0.5;2.5]	1.6 [1.1;2.4]	4.5 [4.0;6.2]^∗^	37.5 [25.1;39.3]^###,^^§§^
	mNTS	5.4 [2.2;6.4]	4.7 [2.5;7.5]	7.7 [5.0;13.0]	105.2 [65.7;129.2]^###,^^§§^
	ROb	3.4 [2.1;3.6]	3.2 [2.9;4.6]	4.8 [2.0;6.3]	17.1 [10.0;26.4]^##,^^§§^
	RPa	9.4 [6.1;11.9]	7.6 [3.7;8.4]	7.7 [4.7;9.1]	16.1 [8.0;22.1]^#,^^§^
	RMg	13.9 [11.7;18.9]	11.8 [10.1;13.6]	13.4 [9.3;19.9]	19.6 [13.1;30.8]
	PP	2.9 [1.5;3.7]	2.7 [1.7;3.1]	5.6 [5.4;7.1]^∗∗^	4.8 [3.7;5.9]^§§^
	VLM	12.6 [11.3;17.4]	12.5 [11.2;18.2]	26.0 [21.4;28.8]^∗∗^	46.5 [36.5;89.4]^##,^^§§^
	RTN	16.2 [9.0;18.6]	14.4 [10.3;18.0]	22.9 [18.9;26.5]^∗^	23.3 [21.8;25.4]^§^
	pFRG	6.2 [2.9;9.5]	4.3 [3.0;5.5]	6.8 [5.5;9.2]	4.6 [3.9;6.6]
Pons	A5	15.7 [9.0;21.3]	14.4 [13.0;16.2]	16.6 [12.8;19.5]	22.7 [17.7;38.4]^§^
	LC	3.2 [1.7;4.2]	0.9 [0.5;1.6]	22.5 [9.0;35.8] ^∗^	81.9 [34.8;88.2]^#,^^§§^
	lPB	2.7 [0.6;3.5]	0.2 [0.0;0.6]	3.7 [0.3;8.8]	7.6 [4.8;12.9]^§§^
	mPB/KF	0.8 [0.4;1.2]	1.4 [0.7;1.9]	11.9 [5.5;16.8]	19.4 [10.5;22.9]^§§^
Mesencephalon	dPAG	0.2 [0.2;0.5]	0.3 [0.1;0.5]	0.2 [0.0;0.7]	0.9 [0.3;1.5]
	dlPAG	0.3 [0.2;1.5]	0.7 [0.1;6.1]	0.9 [0.3;4.7]	0.8 [0.4;1.5]
	lPAG	2.7 [0.9;5.7]	2.3 [1.7;4.5]	5.5 [1.6;10.3]	26.2 [10.8;50.0]^#^
	vlPAG	1.3 [0.5;2.0]	1.2 [0.7;2.6]	6.3 [2.3;10.8]^∗^	25.6 [15.4;33.9]^#^
Diencephalon	DM	28.0 [21.2;31.6]	22.6 [15.1;48.8]	23.9 [19.6;38.0]	23.6 [13.9;48.6]
	LH	28.9 [21.3;29.5]	20.2 [16.8;29.9]	33.9 [29.0;59.7]	23.8 [17.4;42.5]
	PeF	0.8 [0.4;0.9]	0.8 [0.3;0.8]	3.2 [2.1;6.2]^∗^	2.8 [2.5;5.4]
	PH	3.8 [3.7;7.0]	2.6 [0.9;7.0]	15.0 [8.8;20.7]	14.2 [6.5;19.0]

*Values are expressed as median number of c-FOS positive cells per section [Q1;Q3]. Comparison between DMSO and ETO groups under normal pH conditions shows no significant difference. ^∗^ indicates a significant increase in number of c-FOS positive cells per structure under prolonged metabolic acidosis condition compared to normal pH condition both in the presence of DMSO. # indicates a significant increase in number of c-FOS positive cells per structure under prolonged metabolic acidosis condition in the presence of ETO (5⋅10^–1^ μM) compared to that in the presence of DMSO. ^§^ indicates a significant increase in number of c-FOS positive cells per structure under prolonged metabolic acidosis condition compared to normal pH condition in the presence of ETO (5⋅10^–1^ μM). ^∗^*p* < 0.05, ^∗∗^*p* < 0.01, ^#^*p* < 0.05, ^##^*p* < 0.01, ^###^*p* < 0.001, ^§^*p* < 0.05, ^§§^*p* < 0.01. A5: A5 region, cNTS: commissural part of the nucleus tractus solitarius, dlPAG: dorsolateral part of the periaqueductal gray, DM: dorsomedial hypothalamic nucleus, dPAG: dorsal part of the periaqueductal gray, LC: locus coeruleus, LH: lateral hypothalamic area, lPAG: lateral part of the periaqueductal gray, lPB: lateral subnucleus of the parabrachial nucleus, mNTS: median part of the nucleus tractus solitarius, mPB/KF: medial subnucleus of the parabrachial nucleus/kölliker-fuse nucleus, PeF: perifornical region, pFRG: parafacial respiratory group; PH: posterior hypothalamic area, PP: parapyramidal group, RMg: raphe magnus nucleus, ROb: raphe obscurus nucleus, RPa: raphe pallidus nucleus, RTN: retrotrapezoid nucleus; VLM: ventrolateral medullary reticular nucleus, vlNTS: ventrolateral part of the nucleus tractus solitarius, vlPAG: ventrolateral part of the periaqueductal gray.*

#### Prolonged Metabolic Acidosis Induces an Increase in Number of c-FOS Positive Cells in Absence of Etonogestrel

We next investigated whether prolonged metabolic acidosis stimulated *c-fos* expression in brainstem and diencephalic structures. Prolonged metabolic acidosis induces a significant increase in number of c-FOS positive cells relative to that at normal pH at the level of the *medulla oblongata* in vlNTS (*p* < 0.05), PP (*p* < 0.01), VLM (*p* < 0.01) and RTN (*p* < 0.05), pons in LC (*p* < 0.05), mesencephalon in vlPAG (*p* < 0.05), and diencephalon in PeF (*p* < 0.05) (Table 1).

#### Etonogestrel Strengthens Metabolic Acidosis-Induced Increase in Number of c-FOS Positive Cells in Some Brainstem Respiratory Structures

**FIGURE 3:**
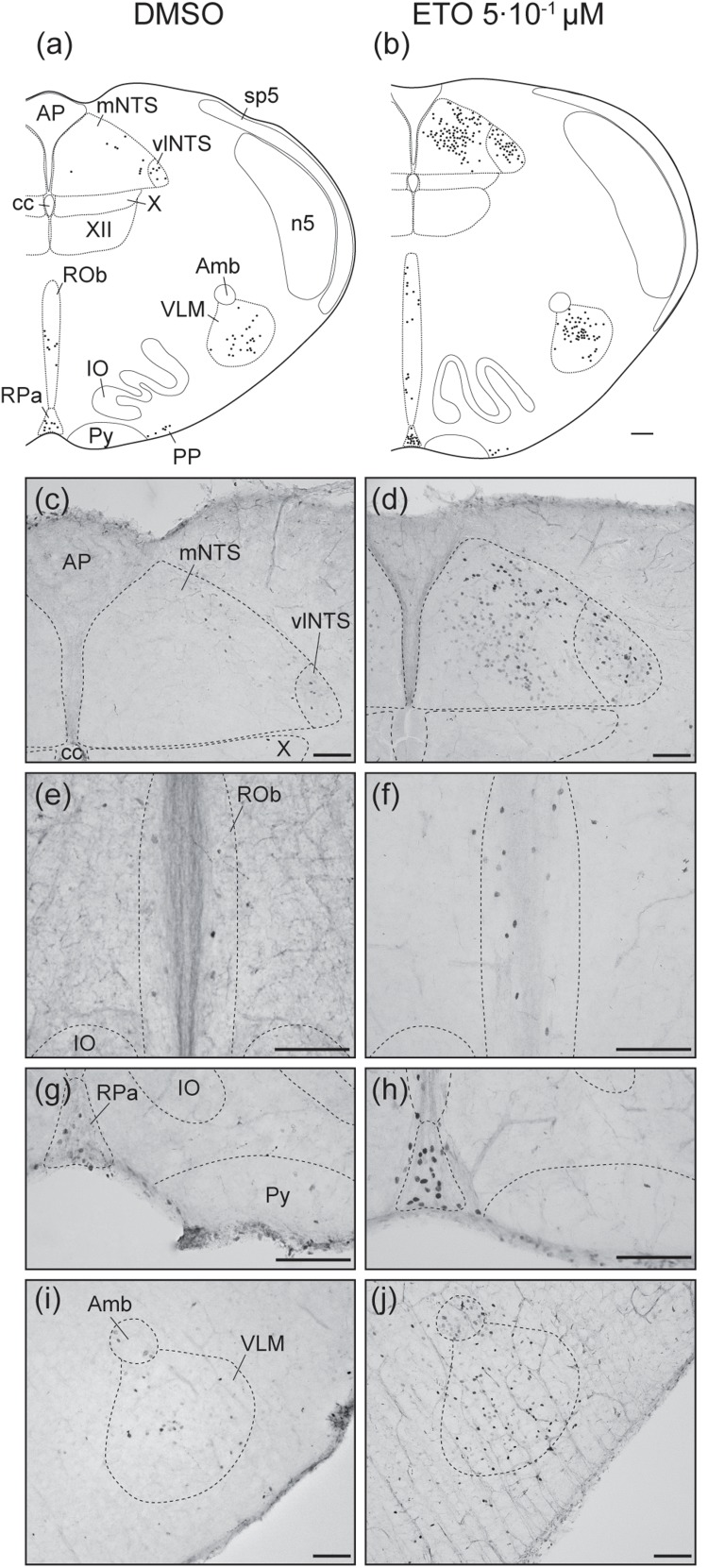
Etonogestrel increases or enhances increase in prolonged metabolic acidosis-induced *c-fos* expression in medullary respiratory-related areas. Drawings representing distribution of cells immunoreactive for c-FOS during prolonged metabolic acidosis (black points) in medulla oblongata under DMSO **(a)** or 5⋅10^–1^ μM etonogestrel **(b)** exposure. Photomicrographs illustrating metabolic acidosis-induced c-FOS immunoreactivity under DMSO **(c,e,g,i)** or etonogestrel **(d,f,h,j)** exposure in mNTS and vlNTS **(c,d)**, ROb **(e,f)**, RPa **(g,h)**, and VLM **(i,j)**. Scale bar = 100 μm. Abbreviations: ambiguus nucleus (Amb), area postrema (AP), central canal (cc), etonogestrel (ETO), hypoglossal nucleus (XII), inferior olives (IO), median part of the nucleus of the tractus solitarius, (mNTS), parapyramidal group (PP), obscurus (ROb) and pallidus (RPa) raphe nuclei, pyramidal tract (Py), ventrolateral medullary reticular nucleus (VLM), ventrolateral part of the nucleus of the tractus solitarius (vlNTS), and dorsal motor nucleus of vagus (X).

**FIGURE 4:**
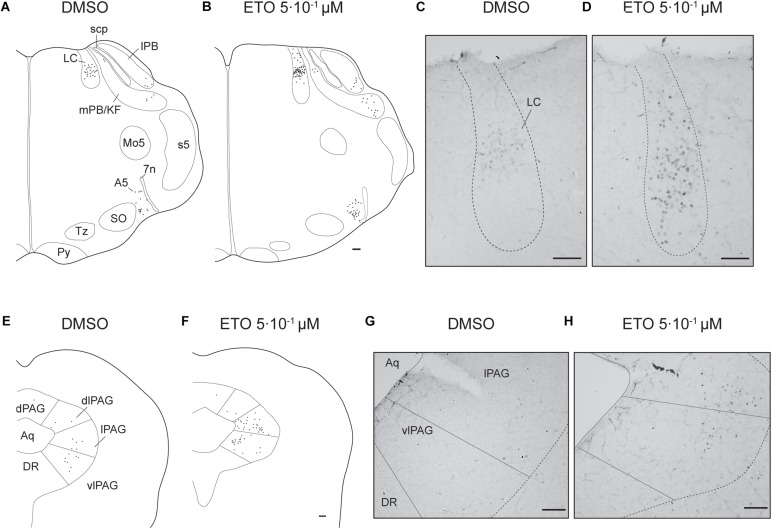
Etonogestrel increases or enhances increase in prolonged metabolic acidosis-induced *c-fos* expression in supra-medullary respiratory-related areas. Drawings representing distribution of cells immunoreactive for c-FOS during prolonged metabolic acidosis (black points) in pons and in mesencephalon under DMSO **(A,C)** or 5⋅10^–1^ μM etonogestrel **(B,D)** exposure. Photomicrographs illustrating metabolic acidosis-induced c-FOS immunoreactivity under DMSO **(E,G)** or 5⋅10^–1^ μM etonogestrel **(F,H)** exposure in LC **(E,F)**, lPAG, and vlPAG **(G,H)**. Scale bar = 100 μm. Abbreviations: A5 region (A5), aqueduct of Sylvius (Aq), dorsolateral part of the periaqueductal gray (dlPAG), dorsomedian part of the periaqueductal gray (dPAG), dorsal raphe nucleus (DR), etonogestrel (ETO), locus coeruleus (LC), lateral part of the periaqueductal gray (lPAG), lateral parabrachial nucleus (lPB), median raphe nucleus (MnR), medial parabrachial nucleus/kölliker-fuse nucleus (mPB/KF), motor trigeminal nucleus (Mo5), superior olives (SO), trapezoid body (TZ), ventrolateral part of the periaqueductal gray (vlPAG), and facial nerve (7n).

Under prolonged metabolic acidosis, exposure of DBS preparations to 5⋅10^–1^ μM ETO further increases number of c-FOS positive cells at the level of *medulla oblongata*, in VLM (*p* < 0.01) and vlNTS (*p* < 0.001), pons in LC (67.63 ± 10.02 vs. 22.40 ± 7.03, *p* < 0.05), and mesencephalon in vlPAG (*p* < 0.05) (Figures 3, 4, and Table 1). Except for vlPAG, this increase in number of c-FOS positive cells under metabolic acidosis between DMSO and ETO parallels with a significant increase in number of c-FOS positive cells under ETO/prolonged metabolic acidosis compared to ETO/normal pH (*p* < 0.01; Table 2).

**TABLE 2:** *c-fos* expression in brainstem and diencephalic structures of DBS *ex vivo* preparations under prolonged metabolic acidosis condition in the presence of almorexant/DMSO and almorexant/etonogestrel.

		**Prolonged metabolic acidosis**	
	
		**Almorexant/DMSO**	**Almorexant/ETO**
		**(n = 4)**	**(n = 4)**
Medulla oblongata	cNTS	29.9 [29.4;33.3]	29.4 [22.4;40.3]
	vlNTS	6.8 [5.1;8.4]	5.3 [3.4;7.5]
	mNTS	5.2 [4.4;9.2]	11.6 [7.5;15.8]
	ROb	5.2 [4.4;9.2]	11.6 [7.5;15.8]^#^
	RPa	11.5 [9.1;13.5]	15.5 [14.2;16.2]^#^
	RMg	21.3 [21.2;22.0]	20.4 [18.4;25.9]
	PP	7.0 [5.6;8.3]	4.3 [2.5;7.1]
	VLM	20.9 [20.0;25.9]	26.8 [24.6;32.5]
	RTN	21.0 [16.4;25.7]	24.5 [21.8;28.8]
	pFRG	6.9 [4.0;8.9]	7.5 ([7.0;8.1]
Pons	A5	11.1 [9.0;15.1]	15.7 [13.4;22.4]
	LC	1.6 [0.4;3.0]	8.5 [3.0;13.5]
	lPB	1.8 [0.4;14.2]	9.9 [4.6;13.6]
	mPB/KF	6.2 [0.6;10.4]	10.2 [5.0;16.4]
Mesencephalon	dPAG	1.0 [0.7;1.8]	0.8 [0.6;1.1]
	dlPAG	0.8 [0.5;0.9]	1.1 [0.6;1.7]
	lPAG	2.1 [1.5;2.8]	2.7 [2.2;3.5]
	vlPAG	2.5 [2.3;3.4]	4.6 [3.0;5.8]
Diencephalon	DM	23.7 [12.6;40.6]	19.1 [13.6;20.2]
	LH	31.3 [15.3;36.4]	19.7 [14.2;28.5]
	PeF	1.8 [1.2;3.3]	2.0 [1.1;2.9]
	PH	9.4 [3.7;16.3]	4.4 [3.3;5.9]

*Values are expressed as median number of c-FOS positive cells per section [Q1;Q3]. # indicates a significant increase in number of c-FOS positive cells under almorexant (5⋅10^–2^ μM)/ETO (5⋅10^–1^ μM) exposure compared to almorexant/DMSO exposure. ^#^*p* < 0.05. A5: A5 region, cNTS: commissural part of the nucleus tractus solitarius, dlPAG: dorsolateral part of the periaqueductal gray, DM: dorsomedial hypothalamic nucleus, dPAG: dorsal part of the periaqueductal gray, LC: locus coeruleus, LH: lateral hypothalamic area, lPAG: lateral part of the periaqueductal gray, lPB: lateral subnucleus of the parabrachial nucleus, mNTS: median part of the nucleus tractus solitarius, mPB/KF: medial subnucleus of the parabrachial nucleus/kölliker-fuse nucleus, PeF: perifornical region, pFRG: parafacial respiratory group; PH: posterior hypothalamic area, PP: parapyramidal group, RMg: raphe magnus nucleus, ROb: raphe obscurus nucleus, RPa: raphe pallidus nucleus, RTN: retrotrapezoid nucleus; VLM: ventrolateral medullary reticular nucleus, vlNTS: ventrolateral part of the nucleus tractus solitarius, vlPAG: ventrolateral part of the periaqueductal gray.*

#### Etonogestrel Induces Increases in Number of c-FOS-Positive Cells Under Prolonged Metabolic Acidosis in Central Structures Not Activated Without Progestin

Exposure to ETO induces a significant increase in number of c-FOS positive cells under prolonged metabolic acidosis relative to DMSO exposure in structures not activated by prolonged metabolic acidosis without progestin at the level of *medulla oblongata* in cNTS (*p* < 0.001), mNTS (*p* < 0.001), RPa (*p* < 0.05), and ROb (*p* < 0.01), pons in LC (*p* < 0.05), mesencephalon in lPAG (*p* < 0.05) and vlPAG (*p* < 0.05) (Figures 3, 4 and Table 1). For all these structures, this increase in number of c-FOS positive cells under metabolic acidosis between DMSO and ETO parallels with a significant increase in number of c-FOS positive cells under ETO/prolonged metabolic acidosis compared to ETO/normal pH (*p* < 0.05 or 0.01; Table 2).

Additionally, we observed a significant increase in number of c-FOS positive cells under ETO/prolonged metabolic acidosis compared to ETO/normal pH in 2 structures not significantly different between DMSO and ETO under metabolic acidosis at the level of the *medulla oblongata* in RTN (*p* < 0.05), and pons in A5 (*p* < 0.05).

#### Etonogestrel Increases Number of c-FOS Positive Orexin Neurons Under Prolonged Metabolic Acidosis

**FIGURE 5:**
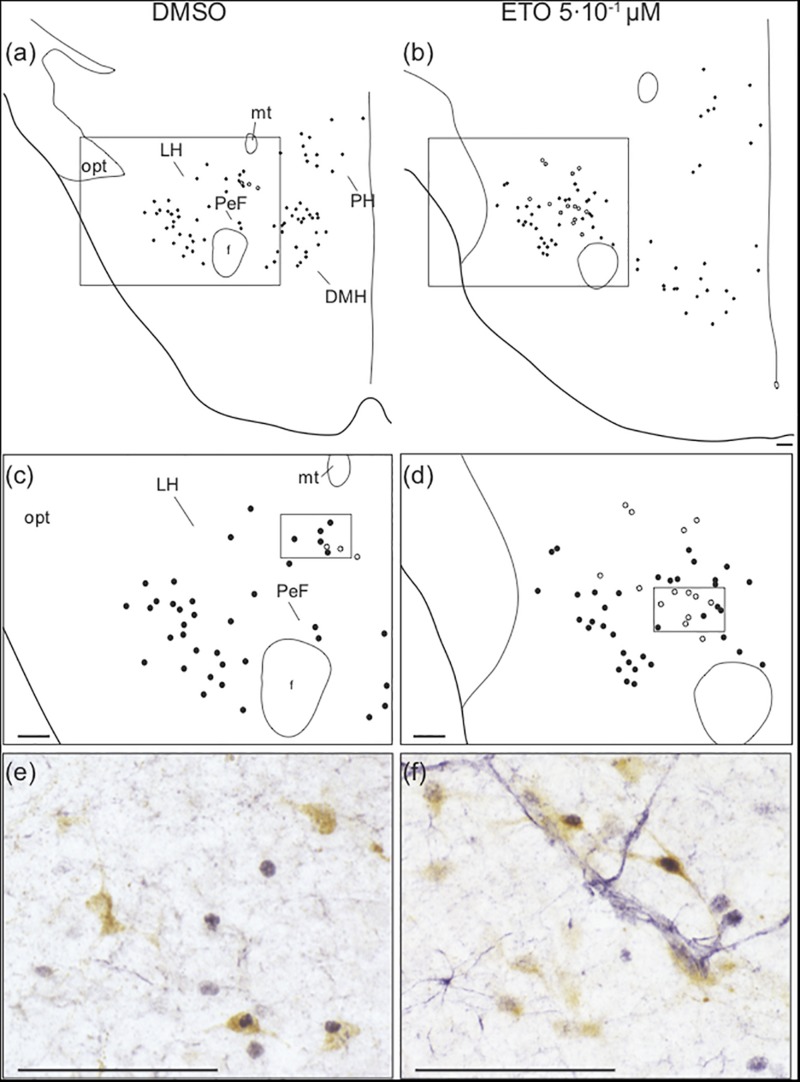
Prolonged metabolic acidosis induced *c-fos* expression was increased in orexin neurons of caudal hypothalamus by etonogestrel. Drawings showing distribution of cells immunoreactive for c-FOS (black points) and both c-FOS and orexin (white points) in caudal hypothalamus under DMSO **(a,c)** or 5⋅10^–1^ μM etonogestrel **(b,d)** exposure. Drawings in panels **(c,d)** represent an enlargement of black rectangle in panels **(a,b)**, respectively. Photomicrographs illustrating metabolic acidosis-induced c-FOS (blue-gray) and orexin (brown) immunoreactivities under DMSO **(e)** and 5⋅10^–1^ μM etonogestrel **(f)** exposure. Photomicrographs correspond to regions outlined by black rectangles in panel **(c)** for **(e)** and in panel **(d)** for **(f)**. Scale bar = 100 μm. Abbreviation: dorsomedian hypothalamic nucleus (DMH), etonogestrel (ETO), fornix (f), lateral hypothalamic area (LH), mamillothalamic tract (mt), optic tract (opt), perifornical area (PeF), posterior hypothalamic area (PH), and third ventricule (V3).

Analysis of the effect of ETO under prolonged metabolic acidosis on various types of *ex vivo* preparations suggests its effect on respiratory response requires the presence of diencephalon. Surprisingly, as stated above, we do not observe any change in number of c-FOS positive cells induced by ETO in caudal hypothalamus, which encompasses DMH, LH, PH, and PeF (Table 1). Orexin neurons are CO_2_/H^+^ chemosensitive (Williams et al., 2007; Song et al., 2012) and project to all brainstem structures displaying an increase or enhanced increase in the number of c-FOS positive cells in our experiments (Peyron et al., 1998; Date et al., 1999; Young et al., 2005; Zheng et al., 2005; Huang et al., 2010; Shahid et al., 2012; Darwinkel et al., 2014). We thus searched for a specific effect of ETO on orexin neurons scattered throughout these areas (Williams et al., 2007). We observe ETO induces an increase in proportion of co-positive neurons for c-FOS and orexin relative to DMSO under prolonged metabolic acidosis in the caudal hypothalamus (7.3 [5.7;12.0] c-FOS/orexin positive cells per section vs. 3.4 [0.9;4.3] c-FOS/orexin positive cells per section, *p* < 0.01; Figure 5).

### Orexin Systems Are Involved in Strengthening of Respiratory Response to Prolonged Metabolic Acidosis Induced by Etonogestrel

We further analyzed the effect of ETO in the presence of almorexant, a specific antagonist of orexin receptors 1 and 2 (OX1R and OX2R, respectively), to further investigate the involvement of orexin systems. We first evaluated orexin’s influence on f_R_ to determine the concentration of almorexant sufficient and necessary to abolish orexin respiratory influence.

#### Orexin A Induces an Increase in f_R_ in DBS Preparations at Normal pH

**FIGURE 6:**
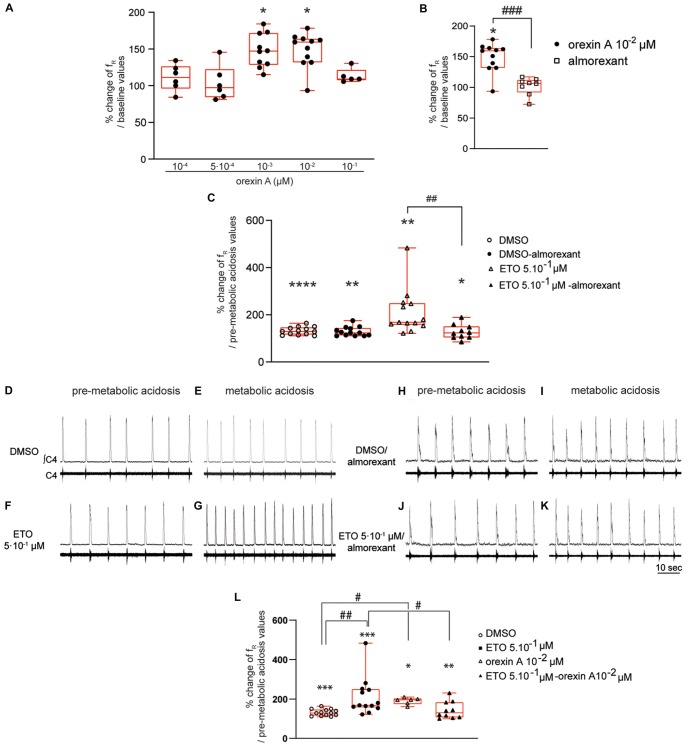
Orexin systems are involved in strengthening of respiratory response to prolonged metabolic acidosis by etonogestrel. Column scatter graph with a superimposed box and whisker plot (median [Q1;Q3]) illustrating percentage of change of f_R_ at 25–30 min of metabolic acidosis at normal pH **(A)** in the presence of orexin at 10^–4^ (*n* = 6), 5⋅10^–4^ (*n* = 6), 10^–3^ (*n* = 10), 10^–2^ (*n* = 11), and 10^–1^ μM (*n* = 5) or **(B)** in the presence of either orexin alone (10^–2^ μM, *n* = 11) or almorexant (10^–2^ μM) and orexin (10^–2^ μM) together (*n* = 8). ^∗^ Indicates a significant change [Wilcoxon signed rank test **(A)** and one-sample *t* test **(B)**] in mean f_R_ relative to baseline values. # Indicates a significant difference [unpaired *t*-test **(B)**] between exposure to orexin alone and co-exposure to almorexant and orexin together. **(C)** histogram showing mean percentage change of f_R_ under conditions of prolonged metabolic acidosis in the presence of either DMSO or ETO alone (DMSO *n* = 13 and ETO *n* = 13) or with almorexant (DMSO *n* = 13 and ETO *n* = 10). ^∗^ Indicates a significant intragroup change (one-sample *t* test) in mean f_R_ relative to values obtained just prior to placing under conditions of metabolic acidosis. # Indicates a significant difference (one-way ANOVA followed by Bonferroni’s multiple comparison test) between groups. Data are expressed as the mean ± SEM.^∗^*p* < 0.05, ^∗∗^*p* < 0.01, ^****^p < 0.0001, ^##^*p* < 0.01, ^###^*p* < 0.001. **(D–K)** Representative traces illustrating respiratory C4 activity recorded in DBS preparations during pre-metabolic acidosis under DMSO **(D)**, ETO **(F)**, DMSO/almorexant **(H)**, or ETO/almorexant **(J)** exposure and during prolonged metabolic acidosis under DMSO **(E)**, ETO **(G)**, DMSO/almorexant **(I)**, or ETO/almorexant **(K)** exposure. **(L)** Column scatter graph with a superimposed box and whisker plot (median [Q1;Q3]) showing percentage change of f_R_ at 25–30 min of metabolic acidosis under conditions of prolonged metabolic acidosis in the presence of either DMSO or ETO alone (DMSO *n* = 13 and ETO *n* = 13) or with orexin (DMSO *n* = 6 and ETO *n* = 10). ^∗^ Indicates a significant intragroup change (Wilcoxon test) in mean f_R_ relative to the values obtained just prior to placing under conditions of metabolic acidosis. # Indicates a significant difference (Kruskal-Wallis test followed by Dunn’s multiple comparisons test) between groups. ^∗^*p* < 0.05, ^∗∗^*p* < 0.01, ^∗∗∗^*p* < 0.001, #*p* < 0.05. ∫C4: integrated activity of the C4 ventral nerve root; C4: electrical activity of the C4 ventral nerve root; ETO: etonogestrel.

Diencephalon-brainstem-spinal cord preparations show a significant increase in f_R_ when exposed to 10^–3^ μM (+47.0% [28.4;71.8], *p* < 0.05) and 10^–2^ μM (+ 59.4% [31.7;63.8], *p* < 0.05) orexin A (Figure 6A). In contrast, 10^–4^ μM (+11.3% [−3.9;26.4]), 5⋅10^–4^ μM (+7.1% [−15.6;22.3]), and 10^–1^ μM (+9.1%% [7.4;21.5]) orexin A does not induce an increase in f_R_ (Figure 6A). We selected 10^–2^ μM orexin A for subsequent experiments to ensure its full effect.

#### Almorexant Completely Blocks Orexin A-Induced Increase in f_R_ of DBS Preparations at Normal pH

Exposing DBS preparations to 5⋅10^–2^ μM almorexant does not induce a change of f_R_ (+10.9% [−7.7;21.8]). At higher concentrations, almorexant exposure significantly increases f_R_ (5⋅10^–1^ μM: +15.4% [3.0;37.5], *p* < 0.05; and 5 μM: +39.9% [5.2;54.5], *p* < 0.01). In light of these results and the results of others (Brisbare-Roch et al., 2007; Li and Nattie, 2010), we performed subsequent experiments involving almorexant in the presence of a concentration of 5⋅10^–2^ μM.

Under antagonization of OX1R and OX2R by almorexant (5⋅10^–2^ μM), 10^–2^ μM orexin A fails to induce significant increase in f_R_ observed in absence of antagonist (+6.3% [−8.2;12.0] vs. +59.4% [31.7;63.8], *p* < 0.001, respectively) (Figure 6B). Exposing DBS preparations to 5⋅10^–2^ μM almorexant was thus considered to be sufficient to abolish the respiratory effect of orexin A.

#### Blocking Orexin Systems by Almorexant Completely Suppresses Strengthening of Respiratory Response to Prolonged Metabolic Acidosis Induced by Etonogestrel

Diencephalon-brainstem-spinal cord preparations superfused with normal pH artificial cerebrospinal fluid (aCSF), containing either almorexant/DMSO or almorexant/5⋅10^–1^ μM ETO, display no change in their f_R_ (+11.3% [−2.6;27.4] and +1.6% [−8.4;14.2], respectively).

In the presence of DMSO, almorexant does not change the increase in f_R_ induced by prolonged metabolic acidosis (Figures 6D,E,H,I); DBS preparations exposed to almorexant/DMSO show a significant increase in f_R_ (+22.7% [12.4;43.7], *p* < 0.01) not significantly different from that observed in absence of almorexant (+29.2% [16.4;46.4]; Figure 6C) at 25–30 min of metabolic acidosis. However, blocking OX1R/OX2R supress strengthening of respiratory response to prolonged metabolic acidosis induced by ETO (Figures 6F,G,J,K); DBS preparations exposed to almorexant/5⋅10^–1^ μM ETO show an increase in f_R_ not significantly different from almorexant/DMSO exposure (+22.8% [3.9;50.4], *p* < 0.05 vs. +22.7% [12.4;43.7]; Figure 6C) at 25–30 min of metabolic acidosis. This increase in f_R_ under almorexant/5⋅10^–1^ μM ETO exposure is significantly lower than under 5⋅10^–1^ μM ETO exposure alone (+68.2% [57.1;149.3], *p* < 0.01; Figure 6C) at 25–30 min of metabolic acidosis.

#### Orexin Strengthens Increase in f_R_ Induced by Prolonged Metabolic Acidosis

In the presence of orexin at 10^–2^ μM, metabolic acidosis induces an increase in f_R_ at 25–30 min of metabolic acidosis (+95.9% [74.5;102.5], *p* < 0.04). This increase in f_R_ is significantly greater than under DMSO alone (+29.2% [16.4;46.4], *p* < 0.02; Figure 6L), and not significantly different from ETO (+68.2% [57.1;149.3]; Figure 6C).

#### Exciting Orexin Systems by Orexin Suppresses Strengthening of Respiratory Response to Prolonged Metabolic Acidosis Induced by Etonogestrel

Under simultaneous orexin/ETO exposure, f_R_ is significantly increased by metabolic acidosis from 15–20 min (+19.2% [7.2;41.9], *p* < 0.05) compared to 25–30 min of metabolic acidosis (+30.0% [6.6;83.8], *p* < 0.04). At the end of metabolic acidosis exposure, increase in f_R_ under orexin/ETO is significantly less from that observed under ETO alone (+68.2% [57.1;149.3]; *p* < 0.05; Figure 6L). To note this response is not significantly different from that observed under DMSO (+29.2% [16.4;46.4]; Figure 6L). Same conclusion is also observed when orexin/ETO co-exposure followed an exposure to orexin alone; DBS preparations exposed to orexin/ETO show an increase in f_R_ induced by metabolic acidosis from 15–20 min (+20.1% [7.4;36.0], *p* < 0.01) that reaches (+21.6% [5.1;42.5], *p* < 0.04) at 25–30 min of metabolic acidosis. This increase in f_R_ is significantly less from that observed under ETO alone (+68.2% [57.1;149.3], *p* < 0.04; Figure 6L), but not significantly different from that observed under orexin alone and DMSO (Figure 6L).

#### Blocking OX1R and OX2R by Almorexant Prevents Increase in *c-fos* Expression Induced by Etonogestrel Under Prolonged Metabolic Acidosis in Most of Structures Modulated by Progestin

Antagonization of orexin signaling suppresses capacity of ETO to increase *c-fos* expression in most of structures displaying an increase or enhanced increase in number of c-FOS positive neurons by the progestin. Indeed, in the presence of almorexant, number of c-FOS positive cells following ETO exposure is no longer statistically different from that observed under DMSO at the level of *medulla oblongata* in cNTS, vlNTS, mNTS, and VLM (27.97 ± 2.14 *vs* 22.27 ± 1.70), pons in LC, and mesensephalon in lPAG and vlPAG (Table 2). In contrast, both RPa and ROb exposed to almorexant/ETO still show a higher number of c-FOS positive cells than when exposed to almorexant/DMSO: ROb (*p* < 0.05), RPa (*p* < 0.05) (Table 2).

Finally, the proportion of orexin immunoreactive cells among population of c-FOS-positive cells in caudal hypothalamus is significantly lower following exposure to almorexant/ETO than ETO alone (2.9 [2.8;3.8] c-FOS/orexin positive cells per section vs. 7.3 [5.7;12.0] c-FOS/orexin positive cells per section, *p* < 0.05, respectively).

## Discussion

This study focused on characterization of central mechanisms underlying the effect of ETO, a progestin of gonane family, on respiratory response to metabolic acidosis. Interest in this progestin arose from a serendipitous clinical observation suggesting desogestrel, the prodrug of ETO, may have been involved in recovery of CO_2_/H^+^ chemosensitivity by CCHS patients (Straus et al., 2010). Here, we obtained data in neonates suggesting this progestin strengthens respiratory response to prolonged metabolic acidosis in a small concentration range and requires presence of the diencephalon by performing electrophysiological recordings of *ex vivo* CNS preparations following application of ETO. Furthermore, we used a functional immunohistochemical approach combined with pharmacological applications to demonstrate orexin systems play a key role in strengthening of both respiratory response to metabolic acidosis and recruitment of respiratory brainstem structures by ETO in the used *ex vivo* CNS preparations.

Prolonged metabolic acidosis increased f_R_, similar to previously described (Kawai et al., 2006; Gestreau et al., 2010), regardless of preparation i.e., MS, PMS, BS and DBS. This augmented f_R_ is paralleled by an increase in number of c-FOS-positive cells in VLM. Increase in *c-fos* expression in this structure is consistent with an increase in CRD as already discussed in contexts other than metabolic acidosis (Okada et al., 2002; Voituron et al., 2005; Joubert et al., 2016). Indeed, VLM encompasses the ventral respiratory group, a neuronal functional unit controlling the CRD (Richter and Spyer, 2001).

Origin of increase in both number of c-FOS-positive cells in VLM and f_R_ may be caused by stimulation of CO_2_/H^+^ chemosensitive cells by metabolic acidosis. Consistent with this hypothesis, our analysis of *c-fos* expression suggests an increase in activity in RTN, considered as an important site of CO_2_/H^+^ chemosensitivity, especially during the neonatal period (Mulkey et al., 2004; Guyenet and Bayliss, 2015; Kumar et al., 2015). In addition to RTN, other medullary and supramedullary structures, including vlNTS, PP, LC, vlPAG, and PeF, displayed increased *c-fos* expression and thus may be involved in the increase in both *c-fos* expression in VLM and f_R_. Indeed, these structures all contain CO_2_/H^+^ chemosensitive neurons and exert an excitatory influence on CRD (Coates et al., 1993; Ryan and Waldrop, 1995; Horn and Waldrop, 1998; Oyamada et al., 1998; Ribas-Salgueiro et al., 2006; Biancardi et al., 2008; Pagliardini et al., 2011; Li N. et al., 2013).

We showed ETO exposure shortened the latency of observing an increase in f_R_ and strengthened the increase in f_R_ induced by prolonged metabolic acidosis on DBS preparations, in accordance with our exploratory study (Loiseau et al., 2014). We only observed this effect in a relatively small concentration range, with a more precocious increase in f_R_ under ETO at 5.10^–2^ and 5.10^–1^ μM than under DMSO and with a significantly more important increase in f_R_ at beginning of metabolic acidosis exposure for ETO at 5.10^–2^ μM and throughout metabolic acidosis exposure for ETO at 5.10^–1^ μM. We consider 5⋅10^–2^ μM is the concentration nearest to that of human exposure, based on the fact CCHS patients who recovered CO_2_/H^+^ chemosensitivity received daily doses of 75 μg desogestrel and considering absolute bioavailability and free fraction of ETO (approximately 74 and 2%, respectively) (Back et al., 1987; Schindler et al., 2003; Straus et al., 2010). However, this concentration is probably underestimated because plasma fraction of progesterone available for transport through blood-brain barrier is not limited to free fraction, but also includes a portion of progesterone-bound fraction (Pardridge and Mietus, 1979). Taking these data into account, we suggest that dose of active ETO in CNS was inappropriate in CCHS patient who did not recover CO_2_/H^+^ chemosensitivity under desogestrel (Li D.C. et al., 2013). In contrast, concentration of progestin in CNS was sufficient to produce an effect in CCHS patients who recovered CO_2_/H^+^ chemosensitivity (Straus et al., 2010). Difference in the progestin effect between CCHS patients may have been associated with differences in ETO metabolism or brain-blood barrier permeability to the steroids. These hypotheses require further investigations, but present data might suggest quantity of desogestrel to be administered will need to be adjusted, case by case, to find effective dose.

We showed ETO induced a strengthening of respiratory response to prolonged metabolic acidosis by interacting with diencephalic, but not brainstem structures. We predict caudal hypothalamic CO_2_/H^+^-stimulated neurons are involved in ETO-induced strengthening of increase in f_R_, because of the existence of CO_2_/H^+^-stimulated neurons in the caudal hypothalamus (Dillon and Waldrop, 1993), a region suggested to be involved in mediating the respiratory effect of progesterone (Bayliss et al., 1990). Analysis of *c-fos* expression revealed ETO led to a significant increase in number of c-FOS/orexin-containing neurons scattered throughout in caudal hypothalamus and for which data from literature favor their involvement (Peyron et al., 1998; Date et al., 1999; Williams et al., 2007; Song et al., 2012; Li N. et al., 2013). We therefore hypothesised orexin neurons constitute a key neuronal diencephalic population required for the ETO-induced strengthening of respiratory response to prolonged metabolic acidosis. Electrophysiological recordings performed on DBS preparations in the presence of almorexant, a specific competitive antagonist of OXR1 and OXR2 previously used *in vivo* to investigate the respiratory-related effect of orexin signaling (Li and Nattie, 2010), confirmed our hypothesis. ETO at 5.10^–1^ μM failed to induce a strengthening of respiratory response to prolonged metabolic acidosis in the presence of almorexant supporting the hypothesis that release of orexin following stimulation of orexin neurons by ETO leads to a strengthening of the response. Interestingly, we also observed that under orexin exposure, ETO at 5.10^–1^ μM did not induce a strengthening of respiratory response to prolonged metabolic acidosis or an earlier response as in the absence of orexin. In addition, as under orexin/ETO co-exposure there was no enhancement of the increase in f_R_ induced by prolonged metabolic acidosis, this suggests the respiratory effect of ETO strongly depends on orexin concentration in tissue and more especially in respiratory structures. Such a hypothesis could explain an absence of strengthening of respiratory response to prolonged metabolic acidosis under high concentration of ETO (2 μM; Figures 1I–K); orexin directly superfused in the bath added to the orexin released following stimulation of orexin neurons by ETO would then lead to a concentration too high for observation of the respiratory effect of the progestin as it may be case when ETO was applied at 2 μM.

An explanatory hypothesis for an absence of ETO effect at high concentration or when ETO at an effective dose was combined with orexin could be activation of mechanisms that obscure the response to metabolic acidosis or desensitization or internalization of OXR, already described in other conditions (Dalrymple et al., 2011). About the last point, which would be quite conceivable with regard to the long-term exposure used in our study, the fact we did not observe an effect of ETO in such conditions at the beginning of metabolic acidosis exposure while we observe one when ETO is applied at 5.10^–2^ and 5⋅10^–1^ μM is not favor of this hypothesis. Indeed, such desensitization could explain a long-term loss of an effect observed at onset of metabolic acidosis, which is not case here. Of course, even if it seems unlikely, such a phenomenon of desensitization or internalization of OXR cannot be totally excluded and further experiments are necessary to definitively invalidate this hypothesis.

Several lines of evidence obtained in adult rat or juvenile mice have emerged showing orexin neurons are CO_2_/H^+^ chemosensitive and contribute to respiratory response to hypercapnia (Williams et al., 2007; Li and Nattie, 2010; Song et al., 2012; Li N. et al., 2013). It has been argued the mechanism responsible for CO_2_/H^+^ chemosensitivity of orexin neurons may involve acid-sensing ion channel 1a (ASIC1a; an H^+^-gated neuronal voltage-insensitive cation channel), because subsequent increase in phrenic nerve discharge observed by focal acidification of LH was abolished both by specific destruction of orexin neurons and presence of an ASIC1a antagonist (Song et al., 2012). A mechanism by which ETO activates orexin neurons is yet to be determined, but is likely related to non-genomic rather than genomic effects, as exposure to ETO was brief (45 min). Expression of cognate membrane receptors of progesterone (progesterone receptor membrane component 1, sigma type 1 receptor or membrane receptors of progesterone) by orexin neurons has never been demonstrated. Furthermore, progesterone and medroxyprogesterone neither improved ventilation nor induced recovery of CO_2_/H^+^ chemosensitivity in CCHS patients, making the hypothesis that ETO exerts its effect *via* cognate progesterone membrane receptors unlikely (Weese-Mayer et al., 1992; Sritippayawan et al., 2002). The effect of ETO may therefore depend on an interaction with receptors to other neurotransmitters. Indeed, considerable evidence shows natural progesterone, its metabolites, and synthetic progestins are allosteric modulators of ligand-gated ion channels belonging to the Cys-loop family (GABA_A_, nicotinic acetylcholine, glycine, and 5HT_3_ receptors), as well as glutamatergic ion channel receptors (NMDA and kainate receptors) (Rupprecht and Holsboer, 1999). It is possible that ETO acts through NMDA receptor because (i) orexin neurons express them (Eyigor et al., 2012), (ii) steroids are described to be allosteric modulators of the NMDA receptor (Park-Chung et al., 1994), (iii) ETO potentiates the NMDA-induced increase in f_R_ (Joubert et al., 2016), and (iv) a functional interaction between ASIC1a and the NMDA receptor has been described (Gao et al., 2005). Thus, ETO may activate orexin neurons by exerting positive allosteric modulation of the NMDA receptor, which leads to strengthening of consequences of ASIC1a stimulation by metabolic acidosis and thus increase in f_R_ induced by aCSF acidification. Of course, further experiments are necessary to validate this possible mechanism for action of ETO.

Of course, it cannot be overlooked data presently obtained were from central nervous system of newborn, and clinical observations in CCHS patients are in adults (Straus et al., 2010; Li D.C. et al., 2013). In addition, it must also be added central nervous system of rodents at birth is immature in comparison with that of humans (Mallard and Vexler, 2015). Although prepro-orexin or orexin mRNA and orexin protein are present before birth since E18, E19 or E20 according to different studies (Yamamoto et al., 2000; Van Den Pol et al., 2001; Steininger et al., 2004), orexin systems, like other diencephalic structures, are immature at birth. Although it is not possible to exclude hypothesis that nature of the stimulus, metabolic acidosis or CO_2_, contributes to the discrepancy between present lack of *c-fos* expression in orexin neurons under acidosis condition without ETO and studies carried out at more advanced stages which concluded to activation of these neurons under hypercapnia (Williams et al., 2007; Li and Nattie, 2010; Song et al., 2012; Li N. et al., 2013), immaturity of orexin neurons may be involved. However, even in a context of immaturity, a profound excitatory influence of orexin on neuronal activity was reported early in development, supporting the fact that orexin systems would exert physiological regulations in neonatal period (Van Den Pol et al., 2001; Steininger et al., 2004). Our histological data suggest orexin neurons are activated by metabolic acidosis under exogenous supply of ETO while they are not in the absence of the progestin, an observation which differs from data obtained at a more advanced stage (Williams et al., 2007; Sunanaga et al., 2009; Li et al., 2016). These observations recall previous work reporting 24 h-milk deprivation did not affect levels of prepro-orexin mRNA at P5 whereas an intraperitoneal administration of leptin caused a significant increase in prepro-orexin mRNA level (Yamamoto et al., 2000). At birth, stimulation of orexin neurons in various physiological situations may require a higher level of excitation than at a more advanced stage of development, level of excitation allowed by ETO in our context or by leptin in works of Yamamoto and collaborators. In such a context, it may be assumed that at an advanced stage of development, the effect of ETO could be enhanced or even present at concentrations for which it is not effective in newborn. Another possibility is that in adult the facilitating effect of ETO may be at origin of a strengthening of activation of orexin neurons under conditions of acidosis. Future experiments at later stages of development would be relevant. They will also permit to explore the respiratory impact of ETO at a developmental stage characterized by a CO_2_/H^+^ respiratory response larger than that observed at birth as previously reported (Bamford et al., 1996; Serra et al., 2001; Wickstrom et al., 2002; Davis et al., 2006).

Respiratory-related brainstem structures in DBS preparations (vlNTS, VLM, LC, and vlPAG) exposed to ETO under prolonged metabolic acidosis displayed an enhanced increase in *c-fos* expression in same way that exposure to ETO potentiated the metabolic acidosis-dependant increase in *c-fos* expression. Several other respiratory-related structures (cNTS, mNTS, RPa, Rob, and lPAG) displayed a *de novo* increase in *c-fos* expression; such an increase was not observed without ETO. It is possible that at least a part of these structures was involved in the reinforcement of respiratory response to prolonged metabolic acidosis induced by ETO. In support of this hypothesis, anatomical and/or functional connections are described between these structures and VLM (Miles, 1983; Ross et al., 1985; Connelly et al., 1989; Dean et al., 1990; Coates et al., 1993; Ryan and Waldrop, 1995; Horn and Waldrop, 1998; Oyamada et al., 1998; Viemari et al., 2004; Zhang et al., 2005; Cao et al., 2006; Li and Nattie, 2006; Biancardi et al., 2008; Ptak et al., 2009; Kobayashi et al., 2010; Depuy et al., 2011). It is thus conceivable that enhanced activation or *de novo* activation of these structures by ETO produced additional excitatory inputs to VLM leading to an enhancement of CRD. Of note, according to our *c-fos* analysis, neurons of RTN and pFRG, which are probably missing in CCHS (Dubreuil et al., 2008; Amiel et al., 2009) did not appear to be involved in the effect of ETO. Altogether, our results may highlight, at least in part, the neuronal pathway used by ETO to induce recovery of CO_2_/H^+^ chemosensitivity in some CCHS patients (Straus et al., 2010).

We assume enhanced activation or activation of NTS, VLM, LC, and PAG neurons relied directly or indirectly on orexin binding to OX1R and OX2R, as we did not observe ETO-induced enhanced increased and *de novo* increase in *c-fos* expression in these structures in the presence of almorexant. In contrast, both ROb, and RPa still showed an increased number of c-FOS positive cells in the presence of almorexant, suggesting ETO may act on these structures, independently of activation of orexin neurons under conditions of prolonged metabolic acidosis. Nevertheless, potential action of ETO on ROb and RPa neurons was not sufficient on its own to induce strengthening of respiratory response to CO_2_/H^+^, since we did not observe an enhancement of the increase in f_R_ induced by prolonged metabolic acidosis in DBS preparations under almorexant and in MS preparations. The fact that despite all ETO exerts a facilitating influence on ROb and RPa neurons must be viewed in light of our recent data showing ETO significantly increases baseline f_R_ in MS preparations of newborn mice, probably through direct activation of the serotoninergic neurons of RPa and ROb (Joubert et al., 2016). Our present observation therefore reinforces conclusions of our recent work that had concluded an ETO interaction with serotoninergic neurons while emphasizing that this interaction is not sufficient to lead to a strengthening of the response to metabolic acidosis.

In conclusion, our results highlight a central mechanism of action through which gonane progestin desogestrel may have induced recovery of CO_2_/H^+^ chemosensitivity in CCHS patients. Collectively, our results obtained on *ex vivo* CNS preparations suggest ETO strengthens respiratory response to CO_2_/H^+^ in neonates at a small concentration range and that, its effect relies mostly on activation of orexin neurons, which activate or enhance activation of several brainstem respiratory-related structures, which in turn may exert a facilitatory influence on the CRD. Our data also suggest activation of ROb and RPa neurons by a pathway, independent of orexin signaling, that is yet to be determined. Combined with our previous work on the medullary pathway involved in the effect of ETO on resting breath (Joubert et al., 2016), this study provides important knowledge about respiratory effects of etonogestrel and first clues of how progestins could constitute a therapeutic solution for CCHS.

## Data Availability Statement

The datasets generated for this study are available on request to the corresponding author.

## Ethics Statement

The animal study was reviewed and approved by Charles Darwin Ethics Committee for Animal Experimentation (Ce5/2011/05; APAFIS#2210-2015100812195835v2).

## Author Contributions

CL contributed to conception of experiments, acquisition and analysis of electrophysiological, pharmacological and immunohistochemical data, figure formatting, data interpretation, discussion of results and implications, and writing of the manuscript. AC performed acquisition and analysis of electrophysiological and pharmacological data. BB acquired of immunohistochemical data. FC performed acquisition and analysis of electrophysiological, pharmacological and immunohistochemical data, figure formatting, discussion of results and implications. LB contributed to conception and supervision of all experiments, acquisition of funding, figure formatting, data interpretation, discussion of results and implications, and writing of the manuscript.

## Conflict of Interest

The authors declare that the research was conducted in the absence of any commercial or financial relationships that could be construed as a potential conflict of interest.
